# Association between *GDF5 +104T/C* polymorphism and knee osteoarthritis in Caucasian and Asian populations: a meta-analysis based on case-control studies

**DOI:** 10.1186/s13018-016-0436-4

**Published:** 2016-09-23

**Authors:** Dong Jiang, Zengtao Hao, Dongsheng Fan, Wen Guo, Pengcheng Xu, Chao Yin, Shuzheng Wen, Jihong Wang

**Affiliations:** 1 Inner Mongolia Medical University, Hohhot, 010000 People’s Republic of China; 2 Department of Hand and Microsurgery II, the Second Affiliated Hospital of Inner Mongolia Medical University, Hohhot, 010030 China

**Keywords:** Meta-analysis, Osteoarthritis, Polymorphism, GDF5

## Abstract

**Background:**

Osteoarthritis (OA) is a degenerative joint disease with a complex genetic background. Variants in growth differentiation factor 5 (*GDF5*) have been reported to be associated with rheumatoid arthritis (RA) in several ethnic populations. The present study aimed to assess the association between the *GDF5 +104T/C* polymorphism and the susceptibility of the knee to OA through a meta-analysis of available case-control studies.

**Methods:**

The PubMed and Science Direct citation databases were used to search electronic literature in order to identify studies published between January 2007 and July 2016 that evaluated the association between the *GDF5 +104T/C* polymorphism and the susceptibility of the knee to OA. Different genetic models were used to assess the pooled and stratified data.

**Results:**

A positive association was found in all pooled studies (OR = 0.808, 95 % CI = 0.754–0.866, *p* < 0.001). Regarding genotypes, significant associations were found using a dominant model (OR = 0.777, 95 % CI = 0.708–0.852, *p* < 0.001), a recessive model (OR = 0.723, 95%CI = 0.623–0.839, *p* < 0.001), and an additive model (*CC* vs *TT* OR = 0.648, 95 % CI = 0.552–0.760, *p* < 0.001; *CC* vs *CT* OR = 0.801, 95 % CI = 0.685–0.936, *p* = 0.005). Meta-analysis data were stratified by ethnicity, and the *GDF5 C* allele was found to be positively associated with OA of the knee in both Caucasians and Asians, as were the *GDF5 TC* and *CC* genotypes. In addition, using an additive model, the *CC* genotype was found to be significantly associated with OA of the knee in both Caucasians and Asians when comparing *CC* vs *TT* genotypes, but not in Caucasians when comparing *TT* vs *CT* genotypes.

**Conclusions:**

Meta-analysis results indicated that the *GDF5 +104T/C* polymorphism is a protective factor for OA among Caucasian and Asian populations.

## Background

Osteoarthritis (OA) is a chronic and progressive condition causing pain and disability worldwide and is regarded as a disease of the entire joint [1, 2]. Obesity, age, and joint injury are the major risk factors for OA of the knee [3, 4]. In addition, heritability studies have shown that genetic components account for approximately half of the risk for development of OA of the knee [5–7]. Understanding the genetic factors that influence progression of the disorder is important for the development of therapies to prevent or attenuate the pathogenesis of knee OA [8].

The genetic background of knee OA likely involves multiple genes that encode proteins with significant functions in the underlying disease process. The growth differentiation factor 5 (*GDF5*) gene contains three introns and is located on chromosome 20q11.2, spanning 21.43 kb from 34042573 to 34021146. The GDF5 protein is encoded by the reverse strand (Entrez Gene, http://www.ncbi.nlm.nih.gov/IEB/Research/Acembly/av.cgi). Proteins encoded by *GDF5* are members of the TGF-beta superfamily and the bone morphogenetic protein (BMP) family. Proteins of this group contain a polybasic proteolytic processing site, which can produce a mature protein with seven conserved cysteine residues [9]. These proteins have been shown to be regulators of cell growth and differentiation in both embryonic and adult tissues. Variants of *GDF5* are associated with chondrodysplasia, acromesomelic dysplasia, and brachydactyly [10–12], indicating that *GDF5* may play a protective role in skeletal development. In addition, the *GDF5 +104T/C* single-nucleotide polymorphism (SNP) in the 5′ untranslated region (UTR) of this gene may result in increased risk of OA. Recently, a number of research groups have reported associations between the *GDF5 +104T/C* SNP and the susceptibility to OA in different ethnic populations. Weak or a complete lack of association in different ethnic populations has been reported by other groups, which may be the result of publication bias, differences in allele frequencies between races, or limited sample sizes.

Although several meta-analyses based on differences in strategies have highlighted a possible association between the *GDF5 +104T/C* SNP and knee OA, all meta-analyses did not distinguish between case-control and cohort studies [13–15]. More recently, several new studies have also reported an association between the *GDF5 +104T/C* SNP and risk of knee OA [16, 17]. In the present study, a meta-analysis was performed to evaluate the contribution of the *GDF5 +104T/C* polymorphism to the susceptibility of the knee to OA in different ethnic populations.

## Methods

### Study identification and selection

The PubMed and Science Direct citation databases were used to search electronic literature to identify studies published between January 2007 and July 2016 that evaluated the association between the *GDF5 +104T/C* polymorphism and the susceptibility of the knee to OA. Combinations of keywords used in the search and entered as Medical Subject Headings (MeSH) were as follows: (“osteoarthritis” or “*GDF5*” or “polymorphism” or “*GDF5 +104T/C*”); (“genetics” or “OA” or “rs143383”); and (“polymorphism” or “polymorphisms”).

### Inclusion and exclusion criteria

Data were collected from fully published articles, excluding any conference or meeting abstracts. Inclusion criteria were as follows: (a) evaluation of the association between *GDF5 rs143383* polymorphism with susceptibility to knee OA; (b) case-controlled study design based on unrelated individuals; and (c) case and control groups having sufficient genotypic data (*T* and *C*) and/or sufficient allelic data (*TT*, *TC*, and *CC*) for estimation of odds ratios (OR) with 95 % confidence interval (95 % CI). Exclusion criteria were as follows: (a) overlapping data; (b) genotype frequencies or numbers not reliably ascertained; (c) cohort or family-based study design, because the design and analysis of the study are based on linkage; and (d) review paper or abstract.

### Data extraction

The following information was extracted: (a) name of the first author, (b) year of publication, (c) ethnicity of the studied population, (d) the numbers of cases and controls of *GDF5* polymorphism, and (e) Hardy-Weinberg equilibrium (HWE) of controls.

### Statistical analysis

Allele frequencies of the *GDF5 +104T/C* polymorphism from each respective study were determined by the allele counting method. HWE was used to examine the deviation of data associated with the *GDF5 rs143383* SNP in the knee OA control groups using Fisher’s exact test. If the *p* value of HWE was not greater than 0.05, the control group was considered to be in disequilibrium. To evaluate the strength of association between the *GDF5 rs143383* polymorphism and the susceptibility of the knee to OA, pooled ORs and their 95 % CIs were determined for each study, and within-study and between-study heterogeneity were evaluated by Cochran’s *Q* statistic [18].

The pooled ORs were performed for allelic contras, dominant, recessive, and additive models. If a *p* value for a particular *Q* statistic was less than 0.10, the random effects model was used [19]. The *I*^2^ measure (*I*^2^ = 100 % *x*(*Q* − *df*)/*Q*) was used to quantify the effect of heterogeneity [20]. *I*^2^ ranges between 0 and 100 %, which, respectively, represents the proportion of inter-study variability attributable to heterogeneity rather than chance (low, 0–25 %; moderate, 25–50 %; large, 50–75 %; and very large, >75 %). When *I*^2^ is greater than 50 %, a study should be eliminated that performs for *I*^*2*^ values to reach less than 25 % [21].

Sensitivity analysis was then performed by excluding studies violating HWE [22].

To evaluate publication bias, funnel plots were determined. However, due to the limited number of studies, Egger’s linear regression test was used to evaluate the bias [23]. When the pooled study groups are homogenous, the random effects and fixed effects models are similar. However, if this is not the case, the random effects model usually provides wider CIs than the fixed effects model [19]. In the present study, statistical manipulations were performed using Stata 11 software (Stata Corporation, College Station, TX).

## Results

**Fig. 1:**
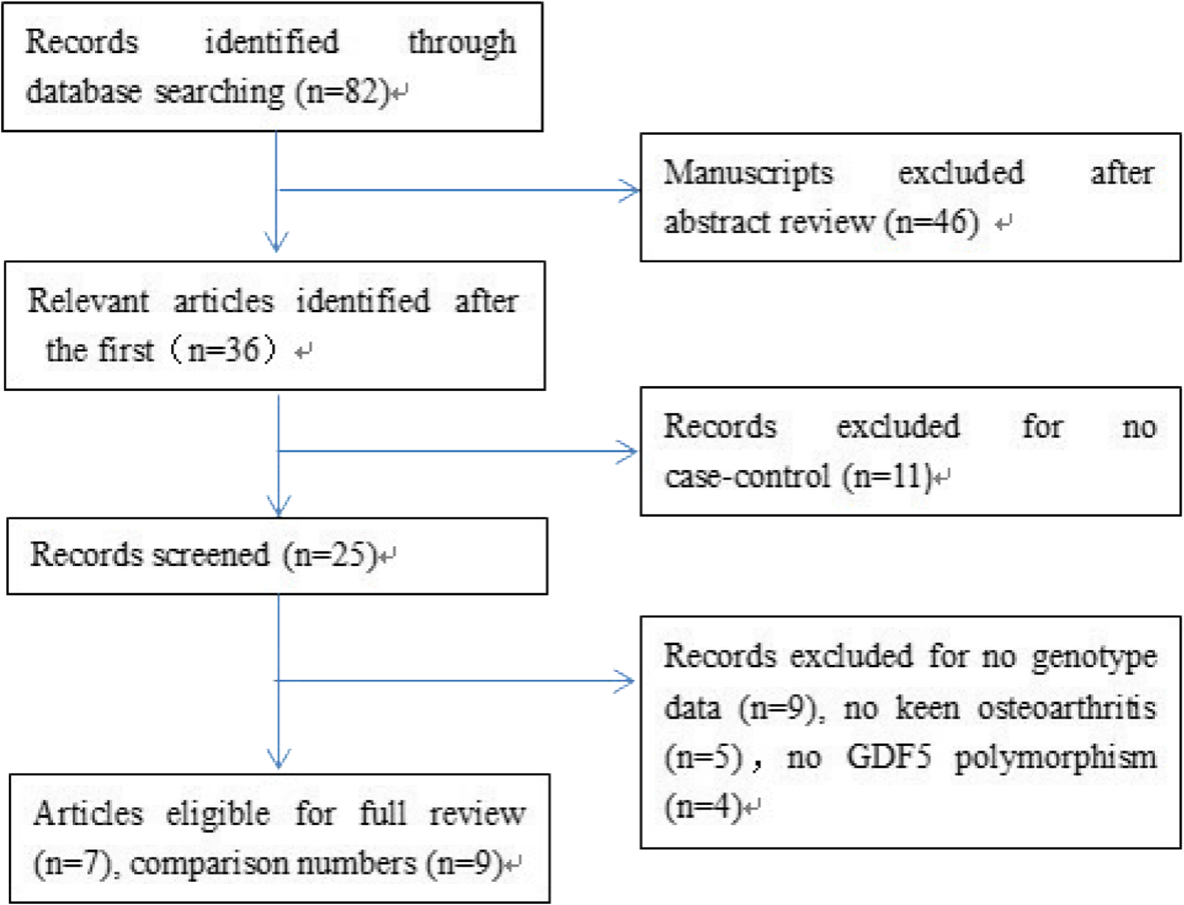
Flow chart of studies of *GDF5 rs143383* polymorphism in the meta-analysis

Fifty-two articles that evaluated the association between *GDF5 +104T/C* polymorphism and the susceptibility of the knee to OA were identified (Fig. 1). Twenty-eight papers were excluded due to data missing, not being a case-control study, genotype distribution in the controls being inconsistent with HWE, previous meta-analysis studies, or missing data. Seven articles met the inclusion criteria [17, 24–29]. Among these, one study reported on two populations (Chinese and Japanese; UK and Spanish; two different populations in the UK) that were considered as two separate studies [24, 25, 28]. The seven identified papers included nine case-control studies involving 3319 knee OA patients and 4987 controls that were conducted in Caucasian and Asian populations.

Characteristics of the *GDF5 +104T/C* studies included in the present meta-analysis are listed in Table 1. The results showed a positive association in all pooled studies (OR = 0.808, 95 % CI = 0.754–0.866, *p* < 0.001) (Table 2). Regarding genotypes, a significant association was found using a dominant model (OR = 0.777, 95 % CI = 0.708–0.852, *p* < 0.001), a recessive model (OR = 0.723, 95 % CI = 0.623–0.839, *p* < 0.001), and an additive model (*CC* vs *TT* OR = 0.648, 95 % CI = 0.552–0.760, *p* < 0.001; *CC* vs *CT* OR = 0.801, 95 % CI = 0.685–0.936, *p* = 0.005). After meta-analysis data were stratified by ethnicity, the *C* allele of *GDF5* was found to be positively associated with knee OA in Caucasians (OR = 0.872, 95 % CI = 0.794–0.957, *p* = 0.004) and Asians (OR = 0.738, 95 % CI = 0.665–0.818, *p* < 0.001). In addition, the GDF5 *TC* and *CC* genotypes were found to be positively associated with knee OA in Caucasians (OR = 0.853, 95 % = 0.749–0.972, *p* = 0.017) and Asians (OR = 0.706, 95 % CI = 0.619–0.806, *p* < 0.001). Using an additive model, the *CC* genotype was found to be significantly associated with knee OA in Caucasians (OR = 0.648, 95 % CI = 0.552–0.760, *p* = 0.007) and Asians (OR = 0.518, 95 % CI = 0.402–0.668, *p* < 0.001) when comparing *CC* vs *TT* genotypes, and when comparing *TT* vs *CT* genotypes (OR = 0.743, 95 % CI = 0.578–0.955, *p* = 0.021), but not significantly associated with knee OA in Caucasians when comparing *TT* vs *CT* genotypes (OR = 0.840, 95 % CI = 0.685–1.026, *p* = 0.088) (Table 2).

**Table 1:** Characteristics of the studies of *GDF5 rs143383* polymorphism included in the meta-analysis

First author	Year	Ethnicity	Numbers		RA/controls (allele)		HWE (*P*)
			OA	Controls	*T*	*C*	
Southam et al.^a^	2007	Caucasian	349	822	450/1020	248/624	0.262
Southam et al.^a^	2007	Caucasian	274	1196	340/1441	208/951	0.550
Miyamoto et al.^a^	2007	Asian	718	861	1131/1276	305/446	0.966
Miyamoto et al.^b^	2007	Asian	313	485	491/681	135/289	0.283
Tsezou et al.	2007	Caucasian	251	268	316/323	186/213	0.669
Valdes et al.	2009	Caucasian	735	654	987/805	483/487	0.320
Cao et al.	2010	Asian	276	298	415/431	137/165	0.360
Tawonsawatruk et al.	2011	Asian	103	103	117/113	63/93	0.424
Mishra et al.	2013	Asian	300	300	378/328	222/272	0.188

Letters ^a^ and ^b^ denote an independent study in one article

*HWE* Hardy-Weinberg equilibrium

**Table 2:** Meta-analysis of the association between *GDF5 rs143383* polymorphism and OA susceptibility

Comparison	Ethnic group	Studies	Sample size		Test of association					Test of heterogeneity		
			OA	Control	OR	95 % CI		*p* value	Model	*Q* test	*p* value	*I* ^2^ (%)
Allelic contrast	Overall	9	3319	4987	0.808	0.754	0.866	0.000	*R*	10.12	0.698	0
(*T* vs *C* allele)	Caucasian	4	1609	2940	0.872	0.794	0.957	0.004		1.43	0.518	0
	Asian	5	1710	2047	0.738	0.665	0.818	0.000		3.25	0.257	21
Dominant model	Overall	9	3319	4987	0.777	0.708	0.852	0.000	*F*	17.1	0.029	53.2
(*TC* + *CC* vs *TT*)	Caucasian	4	1609	2940	0.853	0.749	0.972	0.017		5.99	0.112	49.9
	Asian	5	1710	2047	0.706	0.619	0.806	0.000		7.16	0.128	44.1
Recessive model	Overall	9	3319	4987	0.723	0.623	0.839	0.000	*R*	6.66	0.574	0
(*CC* vs *TC* + *TT*)	Caucasian	4	1609	2940	0.797	0.659	0.965	0.02		1.60	0.659	0
	Asian	5	1710	2047	0.621	0.489	0.789	0.000		2.57	0.633	0
Additive model	Overall	9	3319	4987	0.648	0.552	0.760	0.000	*R*	5.84	0.665	0
(*CC* vs *TT*)	Caucasian	4	1609	2940	0.754	0.613	0.927	0.007		0.15	0.986	0
	Asian	5	1710	2047	0.518	0.402	0.668	0.000		0.69	0.953	0
Additive model	Overall	9	3319	4987	0.801	0.685	0.936	0.005	*R*	9.64	0.291	17
(*CC* vs *TC*)	Caucasian	4	1609	2940	0.84	0.688	1.026	0.088		4.49	0.213	33.2
	Asian	5	1710	2047	0.743	0.578	0.955	0.021		4.63	0.328	13.6

*R* random model, *F* fixed model

### Heterogeneity and publication bias

The between-study heterogeneity in terms of the ORs of the *GDF5 +104T/C* polymorphism were found in all subjects and thus, meta-analysis of the *GDF5 +104T/C* polymorphism was performed using a random effects model for all subjects, with the exception of the dominant model, which was analyzed using a fixed effects model (*I*^2^ = 53.2 %) (Table 2).

**Fig. 2:**
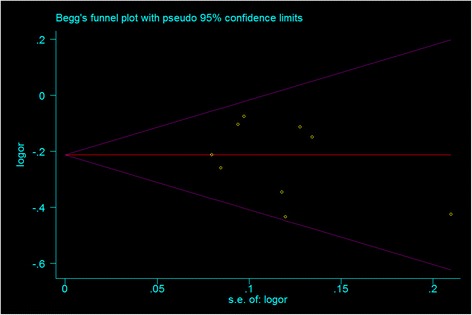
Funnel plot of the meta-analysis of OA risk and the rs143383 polymorphism

A publication bias was identified in the present study (Fig. 2), due to the disproportionate number of articles reporting positive results. Evidence for this was found using recessive and additive models, as the *p* value of Egger’s regression was less than 0.1. In view of this, the “trim and fill” method was used in order to adjust for publication bias in the present study. Adjusted ORs obtained using the “trim and fill” method remained statistically significant (data not shown).

### Sensitivity analyses

The HWE-violating studies were excluded in order to perform sensitivity analyses, and the stability of the results was then evaluated. Departure from HWE was observed in the control of one study (Valdes et al. [28]). After excluding this study, the corresponding ORs did not change substantially in all models, suggesting that the results of the present meta-analysis are stable (data not shown).

## Discussion

The association between the *+104C* allele at the *rs143383* polymorphism in the *GDF5* gene and OA of the knee has been documented in genome-wide association studies (GWAS), with inconsistent results in different case-controls. Meta-analysis is known to be a suitable methodology for detecting small effects in genetic association studies, and the present study was designed in order to update and investigate the results associating the *GDF5 +104T/C* polymorphism with the susceptibility to OA of the knee in different ethnic populations. Seven studies relating to *GDF5 +104T/C* polymorphism were included in the present meta-analysis.

Significant association between the *GDF5 +104T/C* polymorphism and the susceptibility to OA of the knee has been demonstrated in the present study. These results indicate that the *GDF5 rs143383 C* allele was significantly related to OA of the knee in Caucasian and Asian subjects. *GDF5 TC* and *CC* genotypes were found to be significantly related to OA of the knee in Caucasian and Asian subjects. In addition, the *GDF5 rs143383 TT* genotype was found to be significantly related to OA of the knee in Caucasian and Asian subjects, although not significantly related to OA of the knee in Caucasians when evaluated using an additive model (*CC* vs *TC*).

The *GDF5* gene is located on chromosome 20q11.2 and spans 21.43 kb, from 34042573 to 34021146, on the reverse strand. The protein encoded by *GDF5* is a member of the bone morphogenetic protein (BMP) family. *GDF5* gene variants are associated with brachydactyly, chondrodysplasia, and acromesomelic dysplasia, indicating that the *GDF5* gene product plays a critical role in skeletal development [30]. Several animal studies have confirmed data supportive of a key role for *GDF5* [31–33]. In animals with *GDF5* variants, multiple joint abnormalities have been reported, including tendon anomaly, soft tissue deformities, and decreases in the appendicular skeleton. Overall, these results demonstrate that variants of *GDF5* may play a crucial role in the pathogenesis of OA.

Heterogeneity in different ethnic populations is a potential problem that is further complicated by allelic frequencies of different susceptibility genes [34]. In the present study, significant heterogeneity was identified among different ethnic groups. To evaluate this further, ethnicity was used to stratify the studies included in order to clarify the heterogeneity. The results indicated that part of the heterogeneity identified was attenuated. In addition, every study with the unique criteria defined the cases, which may have resulted in the observed heterogeneity. Some of the studies used in the evaluation used the ACR criteria and/or K/L classification to define the respective cases of OA, while other studies defined OA using the TKR criteria. These differences between the control groups and the key characteristics of the participants may also result in the observed heterogeneity in the magnitude of the genetic effects [35]. Recruiting a matched control group may contribute to the magnitude of heterogeneous genetic effects [35]. Additional factors may also be taken into consideration in order to identify heterogeneity in the event additional data were available.

Previous meta-analysis studies have reported an association between *GDF5 +104T/C* polymorphism and the susceptibility to OA of the knee in Asians and Caucasians. These studies also reported that the *GDF5 rs143383 C* allele serves as a protective factor for OA in Caucasians (OR = 0.87, *p* < 0.001) and Asians (OR = 0.78, *p* = 0.003) [14]. Rui et al. reported an association between *CDF5 rs143383* polymorphism and knee OA, hip OA, and hand OA using random or fixed effects models [13], although the findings did not distinguish between case-control and cohort studies, further complicating the heterogeneity among different studies.

In the present meta-analysis, the incidence of the *GFD5 C* allele was found to vary among different ethnicities, from 19.8 % in Asian to 23.3 % in Caucasian populations. In terms of controls, the frequencies of the *GDF5 C* allele in the Asian and European populations were 28.1 and 45.7 %, respectively. Although a comprehensive meta-analysis was performed, some limitations should be acknowledged in the present study. Some studies were excluded in spite of establishing search criteria, as raw data is insufficient. In addition, between-study heterogeneity and publication bias may influence the results in the present meta-analysis.

## Conclusions

In conclusion, the meta-analysis confirmed that the *GDF5 +104T/C* polymorphism can confer susceptibility to knee OA with a protective association in the subjects.

## Abbreviations

BMP: bone morphogenetic protein
OA: osteoarthritis
GDF5: growth differentiation factor 5
CIs: confidence intervals
SNP: single-nucleotide polymorphism
UTR: untranslated region
MeSH: Medical Subject Headings
HWE: Hardy-Weinberg equilibrium
GWAS: genome-wide association studies

## References

[1] Murphy L, Helmick CG (2012). The impact of osteoarthritis in the United States: a population-health perspective. Am J Nurs.

[2] O'Conor CJ, Ramalingam S, Zelenski NA, Benefield HC, Rigo I, Little D, Wu CL, Chen D, Liedtke W, McNulty AL, Guilak F (2016). Cartilage-specific knockout of the mechanosensory ion channel TRPV4 decreases age-related osteoarthritis. Sci Rep.

[3] Guilak F (2011). Biomechanical factors in osteoarthritis. Best Pract Res Clin Rheumatol.

[4] van Tunen JA, Dell'Isola A, Juhl C, Dekker J, Steultjens M, Lund H (2016). Biomechanical factors associated with the development of tibiofemoral knee osteoarthritis: protocol for a systematic review and meta-analysis. BMJ Open.

[5] Hochberg MC, Yerges-Armstrong L, Yau M, Mitchell BD (2013). Genetic epidemiology of osteoarthritis: recent developments and future directions. Curr Opin Rheumatol.

[6] Loughlin J (2005). The genetic epidemiology of human primary osteoarthritis: current status. Expert Rev Mol Med.

[7] MacGregor AJ, Li Q, Spector TD, Williams FM (2009). The genetic influence on radiographic osteoarthritis is site specific at the hand, hip and knee. Rheumatology (Oxford).

[8] Vincent TL (2013). Targeting mechanotransduction pathways in osteoarthritis: a focus on the pericellular matrix. Curr Opin Pharmacol.

[9] Martinez-Garcia M, Garcia-Canto E, Fenollar-Cortes M, Aytes AP, Trujillo-Tiebas MJ (2015). Characterization of an acromesomelic dysplasia, Grebe type case: novel mutation affecting the recognition motif at the processing site of GDF5. J Bone Miner Metab.

[10] Al-Qattan MM, Al-Motairi MI, Al Balwi MA (2015). Two novel homozygous missense mutations in the GDF5 gene cause brachydactyly type C. Am J Med Genet A.

[11] Farooq M, Nakai H, Fujimoto A, Fujikawa H, Kjaer KW, Baig SM, Shimomura Y (2013). Characterization of a novel missense mutation in the prodomain of GDF5, which underlies brachydactyly type C and mild Grebe type chondrodysplasia in a large Pakistani family. Hum Genet.

[12] Khan S, Basit S, Khan MA, Muhammad N, Ahmad W (2016). Genetics of human isolated acromesomelic dysplasia. Eur J Med Genet.

[13] Zhang R, Yao J, Xu P, Ji B, Luck JV, Chin B, Lu S, Kelsoe JR, Ma J (2015). A comprehensive meta-analysis of association between genetic variants of GDF5 and osteoarthritis of the knee, hip and hand. Inflamm Res.

[14] Pan F, Tian J, Winzenberg T, Ding C, Jones G (2014). Association between GDF5 rs143383 polymorphism and knee osteoarthritis: an updated meta-analysis based on 23,995 subjects. BMC Musculoskelet Disord.

[15] Liu J, Cai W, Zhang H, He C, Deng L (2013). Rs143383 in the growth differentiation factor 5 (GDF5) gene significantly associated with osteoarthritis (OA)-a comprehensive meta-analysis. Int J Med Sci.

[16] Xiao JL, Meng JH, Gan YH, Zhou CY, Ma XC (2015). Association of GDF5, SMAD3 and RUNX2 polymorphisms with temporomandibular joint osteoarthritis in female Han Chinese. J Oral Rehabil.

[17] Mishra A, Sanghi D, Maurya SS, Singh A, Srivastava RN, Sharma C, Raj S, Avasthi S, Parmar D (2013). Association of polymorphism in growth and differentiation factor 5 gene with osteoarthritis knee. American Journal of Biochemistry & Biotechnology.

[18] Cochran WG (1954). The combination of estimates from different experiments. Biometrics.

[19] Dersimonian R, Nan L (1986). Meta-analysis in clinical trials. Control Clin Trials.

[20] Higgins JPT, Thompson SG (2002). Quantifying heterogeneity in a meta-analysis. Stat Med.

[21] Patsopoulos NA, Evangelou E, Ioannidis JP (2008). Sensitivity of between-study heterogeneity in meta-analysis: proposed metrics and empirical evaluation. Int J Epidemiol.

[22] Thakkinstian A, McElduff P, D'Este C, Duffy D, Attia J (2005). A method for meta-analysis of molecular association studies. Stat Med.

[23] Song F, Gilbody S (1998). Bias in meta-analysis detected by a simple, graphical test. Increase in studies of publication bias coincided with increasing use of meta-analysis. BMJ.

[24] Miyamoto Y, Mabuchi A, Shi D, Kubo T, Takatori Y, Saito S, Fujioka M, Sudo A, Uchida A, Yamamoto S, Ozaki K, Takigawa M, Tanaka T, Nakamura Y, Jiang Q, Ikegawa S (2007). A functional polymorphism in the 5′ UTR of GDF5 is associated with susceptibility to osteoarthritis. Nat Genet.

[25] Southam L, Rodriguez-Lopez J, Wilkins JM, Pombo-Suarez M, Snelling S, Gomez-Reino JJ, Chapman K, Gonzalez A, Loughlin J (2007). An SNP in the 5′-UTR of GDF5 is associated with osteoarthritis susceptibility in Europeans and with in vivo differences in allelic expression in articular cartilage. Hum Mol Genet.

[26] Tawonsawatruk T, Changthong T, Pingsuthiwong S, Trachoo O, Sura T, Wajanavisit W (2011). A genetic association study between growth differentiation factor 5 (GDF 5) polymorphism and knee osteoarthritis in Thai population. J Orthop Surg Res.

[27] Tsezou A, Satra M, Oikonomou P, Bargiotas K, Malizos KN (2008). The growth differentiation factor 5 (GDF5) core promoter polymorphism is not associated with knee osteoarthritis in the Greek population. J Orthop Res.

[28] Valdes AM, Spector TD, Doherty S, Wheeler M, Hart DJ, Doherty M (2009). Association of the DVWA and GDF5 polymorphisms with osteoarthritis in UK populations. Ann Rheum Dis.

[29] Cao Z, Lee HS, Song JH, Yoon JW, Yong KP, Nam SW, Lee JY, Park WS (2010). Growth differentiation factor 5 (GDF5) core promoter polymorphism is not associated with susceptibility to osteoarthritis of the knee in the Korean population. Korean J Pathol.

[30] Francis-West PH, Abdelfattah A, Chen P, Allen C, Parish J, Ladher R, Allen S, MacPherson S, Luyten FP, Archer CW (1999). Mechanisms of GDF-5 action during skeletal development. Development.

[31] Gallagher JA, Ranganath LR, Boyde A (2015). Lessons from rare diseases of cartilage and bone. Curr Opin Pharmacol.

[32] Reynard LN, Loughlin J (2013). The genetics and functional analysis of primary osteoarthritis susceptibility. Expert Rev Mol Med.

[33] Ohta Y, Okabe T, Larmour C, Di Rocco A, Maijenburg MW, Phillips A, Speck NA, Wakitani S, Nakamura T, Yamada Y, Enomoto-Iwamoto M, Pacifici M, Iwamoto M (2015). Articular cartilage endurance and resistance to osteoarthritic changes require transcription factor Erg. Arthritis Rheumatol.

[34] Lee M, Aggen SH, Otowa T, Castelao E, Preisig M, Grabe HJ, Hartman CA, Oldehinkel AJ, Middeldorp CM, Tiemeier H, Hettema JM (2016). Assessment and characterization of phenotypic heterogeneity of anxiety disorders across five large cohorts. Int J Methods Psychiatr Res.

[35] Evangelou E, Chapman K, Meulenbelt I, Karassa FB, Loughlin J, Carr A, Doherty M, Doherty S, Gomez-Reino JJ, Gonzalez A, Halldorsson BV, Hauksson VB, Hofman A, Hart DJ, Ikegawa S, Ingvarsson T, Jiang Q, Jonsdottir I, Jonsson H, Kerkhof HJ, Kloppenburg M, Lane NE, Li J, Lories RJ, van Meurs JB, Nakki A, Nevitt MC, Rodriguez-Lopez J, Shi D, Slagboom PE, Stefansson K, Tsezou A, Wallis GA, Watson CM, Spector TD, Uitterlinden AG, Valdes AM, Ioannidis JP (2009). Large-scale analysis of association between GDF5 and FRZB variants and osteoarthritis of the hip, knee, and hand. Arthritis Rheum.

